# CHO Cell-Produced Truncated Bovine Ephemeral Fever Virus Glycoprotein as a Promising Subunit Vaccine Candidate for Cattle

**DOI:** 10.3390/vaccines14030265

**Published:** 2026-03-15

**Authors:** Huan-Yu Hsu, Shu-Ju Yeh, Chi-Chih Chen, Guan-Ming Ke

**Affiliations:** 1 International Degree Program in Animal Vaccine Technology, International College, National Pingtung University of Science and Technology, Neipu, Pingtung 91201, Taiwan; ariel0715@gmail.com; 2 Graduate Institute of Animal Vaccine Technology, College of Veterinary Medicine, National Pingtung University of Science and Technology, Neipu, Pingtung 91201, Taiwan; workingaja0115@gmail.com; 3 Research Center for Animal Biologics, College of Veterinary Medicine, National Pingtung University of Science and Technology, Neipu, Pingtung 91201, Taiwan

**Keywords:** bovine ephemeral fever, glycoprotein, subunit vaccine, cattle

## Abstract

Background/Objectives: Bovine ephemeral fever (BEF) is a significant disease affecting the cattle industry. The current control strategy for BEF in the field primarily relies on inactivated vaccines. However, some individuals have experienced hypersensitive reactions to these vaccines, prompting the exploration of subunit vaccines as a potential alternative for BEF prevention. Glycoprotein (G protein)-based subunit vaccines derived from virions have successfully induced neutralizing antibodies in cattle for over a decade. Nevertheless, the lack of recent studies evaluating their efficacy using recombinant proteins has raised concerns regarding the development of BEF subunit vaccines for practical field application. Therefore, the objective of this study was to evaluate the antigenicity of a novel truncated G protein produced in mammalian cells as a candidate subunit vaccine for BEF in cattle. Methods: In this study, the G protein with full ectodomain and a version truncated at the C-terminal domain were successfully generated using the ExpiCHO™ expression system. Vaccine efficacy was evaluated weekly by measuring neutralizing antibody titers and cytokine mRNA expression levels following vaccination. Results: Results show that the recombinant protein s510, derived from the G protein of BEF, can stimulate cattle to produce an average 35-fold increase in neutralizing antibodies after three doses of vaccination. The significant upregulation of IFN-γ mRNA supports the effectiveness of the s510-based subunit vaccine and indicates the activation of a cytotoxic immune response in cattle following vaccination. Conclusions: In conclusion, the results indicate that the recombinant protein s510 is a promising antigen for future BEF subunit vaccine development in this study.

## 1. Introduction

Bovine ephemeral fever (BEF) is an arthropod-borne disease primarily transmitted by biting midges (*Culicoides* spp.) and mosquitoes, posing a significant threat to the cattle industry. BEF causes the sudden onset of recurrent fever in cattle, accompanied by salivation, muscle stiffness, joint pain, lameness, recumbency, and in severe cases, even death [1]. Infected cattle often exhibit reduced appetite due to these characteristic clinical symptoms, leading to the loss of body condition in beef cattle and decreased milk quality and productivity in dairy cows. Sudden drops in milk production, loss of body condition, and mortality are common direct causes of economic losses in dairy and beef herds affected by BEF [2,3].

Taiwan has experienced an increase in the frequency of BEF epidemics, with outbreaks occurring every 1–2 years compared to the previous intervals of 5–6 year gaps [2]. While past studies typically reported low mortality rates, recent outbreaks have shown alarmingly high case-fatality rates, sometimes exceeding 20% [4,5]. Mortality can result directly from the disease or from secondary complications such as starvation or pneumonia. In Japan, a 29% mortality rate among beef cattle during outbreaks in 2015 drew significant attention from farmers and animal health agencies, suggesting enhanced pathogenesis of Bovine Ephemeral Fever Virus (BEFV) [6]. Turkey has reported BEF outbreaks in its southern and southeastern regions every few years since 1996, with cases documented in 1999, 2003, 2008, 2012, and 2020, indicating increased frequency [4,7]. The onset of BEF generally coincides with the proliferation of its transmitting vectors, which typically thrive in warm, humid weather [8]. The shortened intervals between BEF outbreaks may be influenced by climate change, including warmer temperatures and altered monsoon patterns. These climate changes may affect the distribution, abundance, and activity periods of the insect vectors responsible for BEFV outbreaks, resulting in an expanded geographical range and an extended active season for these vectors. This could potentially lead to BEFV emergence in new areas or longer transmission seasons in endemic regions [9].

The control strategy of BEF in livestock involves a combination of strategies, including vaccination, transmitted vector control, and potentially managing animal movements. Regarding vaccination, only inactivated vaccines for BEF are currently available on the market. Inactivated vaccines help control BEF by inducing neutralizing antibody responses [10]. However, the efficacy of most inactivated BEF vaccines can vary, and some individuals in Taiwan have experienced adverse effects following vaccination, such as fever, reduced appetite, and prolonged milk production declines in dairy cows, raising concerns about the use of these vaccines. The adaptation of the virus to cell culture and potential epitope alterations caused by the inactivation process also pose challenges for the manufacture of effective inactivated vaccines. Due to these limitations, subunit vaccines have been explored as a potential alternative for BEF prevention [11].

BEFV is an enveloped, bullet-shaped rhabdovirus with a single-stranded, negative-sense RNA genome approximately 14.9 kilobases in length. BEFV comprises five structural proteins: large RNA-dependent RNA polymerase, nucleoprotein, phosphoprotein, glycoprotein, and matrix protein. Among these, the glycoprotein (G protein) serves as the primary neutralizing and protective antigen and has been identified as a potential subunit vaccine candidate due to its ability to induce protective immune responses in mice [12], guinea pigs [13], and cattle [14,15]. In the current subunit vaccine design strategy, the mammalian cell expression system is favored due to its primary advantage of producing antigens with the correct glycosylation pattern derived from viral pathogens. Post-translational modifications in mammalian cells generate glycosylation patterns on antigens that closely resemble those of the native viral proteins. This accurate glycosylation is essential for maintaining the proper antigenic structure of the protein and is critical for eliciting a protective immune response. Notably, mammalian cells produce compositionally more complex N-glycans containing terminal sialic acids, unlike insect cell systems, which typically generate simpler N-glycans [16]. However, the challenges of the mammalian cell expression system include low protein yield and potential variability in the antigenicity of the modified proteins, especially viral glycoproteins [17]. To evaluate the antigenicity of the truncated G protein of BEFV, a recombinant G protein-based subunit vaccine was administered to mice [12] and guinea pigs [13]. After two doses of vaccination, the administration of the truncated G protein successfully elicited antibody responses in these animals, demonstrating the potential of using mammalian cell-produced truncated G protein in a BEF subunit vaccine.

However, there is limited research directly evaluating the efficacy of mammalian cell-produced BEF subunit vaccines in cattle models. The virion G protein combined with the Quil A adjuvant has been shown to enhance the effectiveness of the BEF subunit vaccine in cattle, as demonstrated in a 1994 study [14]. Additionally, the glycoprotein produced by recombinant vaccinia viruses was used to vaccinate cattle in Australia, successfully inducing high titers of neutralizing antibodies against experimental BEFV infection in 1996 [15]. They demonstrate the antigenicity of the original viral G protein, which was derived either from the virus virion or from a recombinant vaccinia virus, but not from mammalian cell culture. To the best of our knowledge, no recent research since that 1996 study has evaluated the efficacy of G protein-based subunit vaccines in cattle. This gap may be primarily due to the low productivity of recombinant G protein produced by cell culture and the challenges associated with obtaining cattle under experimental conditions for subunit vaccine efficacy evaluation. Therefore, the objective of this study was to evaluate the antigenicity of a novel truncated G protein produced in mammalian cells as a candidate subunit vaccine for BEFV in cattle.

In this study, a G protein-based subunit vaccine was successfully produced and administered to three cattle three doses at two-week intervals, resulting in an average 35-fold increase in neutralizing antibody titer on Day 42, which is two weeks after the third vaccination. The antigen for this subunit vaccine was selected from four recombinant proteins produced using the CHO cell expression system, based on the highest productivity and purity. These four recombinant proteins were derived from the G protein of BEFV and included the full ectodomain as well as a version truncated at the C-terminal domain, which is located away from the conformational neutralizing sites. The truncated G protein, fused with secretory signal peptides to enhance purity, was produced with the highest yield and purity and used as the antigen in the subunit vaccine for this study.

## 2. Materials and Methods

### 2.1. Vector Construct and Protein Expression

**Figure 1:**
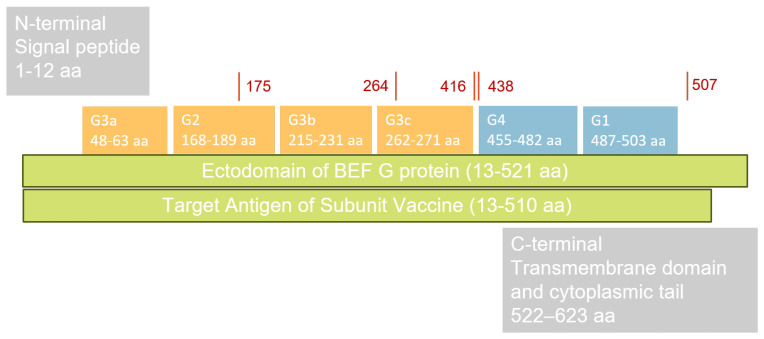
The functional sites of the glycoprotein (G protein) of BEFV are as follows. The ectodomain spans amino acids 13 to 521 (13–521 aa). There are four distinct neutralization sites on the BEFV G protein, designated G1, G2, G3, and G4. The antigenic sites G2 and G3 are conformational neutralizing sites located at the N-terminal and are indicated by orange blocks, whereas antigenic sites G1 and G4 are linear neutralizing sites located at the C-terminal domain and are indicated by blue blocks. Glycosylation sites at amino acids 175, 264, 416, 438, and 507 are labeled in red. The original G protein of BEFV, which includes a signal peptide (amino acids 1 to 12) and a transmembrane domain with a cytoplasmic tail (amino acids 522 to 623), was considered unnecessary for this experiment and therefore excluded. The abbreviation aa represents amino acid.

In this experiment, four distinct recombinant proteins derived from the BEFV G protein were expected for production. The BEFV G protein contains four distinct neutralization sites, designated G1, G2, G3, and G4 [18,19,20]. To generate the antigen, all four neutralization sites (G1–G4) were incorporated during protein production. To ensure the recombinant protein closely mimics the native structure, glycosylation sites at amino acid positions 175, 264, 416, 438, and 507 were also included. The DNA sequence of the BEFV G protein gene was obtained from the most recent outbreak in Taiwan in 2024 and identified as most similar to the China CQ1 strain (GenBank: OP887034.1). Target amino acid sequences corresponding to residues 13–510 (r510 and s510) and 13–521 (r521 and s521) of the China CQ1 strain were selected as the vaccine candidates for this study. The full ectodomain, spanning amino acids 13 to 521, designated r521, was selected as one recombinant protein construct in this study. To improve recombinant protein yield, a truncated version comprising amino acids 13 to 510, designated r510, with deletions at the C-terminus of the BEFV G protein, was chosen as a second construct (Figure 1). To enhance the purity of the recombinant proteins, a secretory signal peptide was reinserted into the expression vectors, named s510 and s521, resulting in the production of four distinct proteins. The sequence was optimized for expression in CHO cells, ligated with a 6× Histidine Tag into the pcDNA 3.4 expression vector, and used to produce the recombinant protein with the ExpiCHO™ Expression System following the manufacturer’s instructions (Gibco™, Thermo Fisher Scientific Inc., Waltham, MA, USA).

### 2.2. Protein Purification and Quantification

The supernatant of cell lysates was filtered by a 0.45 μm PES membrane and purified by immobilized metal-ion affinity chromatography (ӒKTA pure™, Cytiva biotechnology, Danaher corporation, Washington, DC, USA) with a nickel-nitrilotriacetic acid resin column (HisTrap™ HP 5 mL column, Cytiva biotechnology, Danaher corporation, Washington, DC, USA). The target protein was eluted by increasing the imidazole concentration from 20 mM to 500 mM during the purification process. Subsequently, an ultrafiltration process was performed to concentrate the target protein using Amicon^®^ Ultra-15 (Merck Millipore, Merck group, Darmstadt, Germany) with a 30K MWCO membrane, and the buffer was desalted by replacing it with phosphate-buffered saline (PBS). The concentration of recombinant proteins was determined using the extrapolation test with standard bovine serum albumin (BSA) in 10% sodium dodecyl sulfate-polyacrylamide gel electrophoresis (SDS-PAGE). The peak volume of the standard BSA was identified and the volume of the target proteins was also measured at 70 kilo Dalton (kDa), which corresponds to the expected size of the recombinant antigen. After linearizing the BSA volumes, the expression level of the target proteins was determined. The recombinant protein, characterized by high productivity and purity in the 10% SDS-PAGE gel, was selected as the antigen for the BEF subunit vaccine in the subsequent experiment.

### 2.3. Protein Verification by Western Blotting Assay

After completing SDS-PAGE, the gel was transferred onto a 0.22 μm PVDF membrane (Cytiva Biotechnology, Danaher Corporation, Washington, DC, USA) using the Trans-Blot^®^ SD Semi-Dry Transfer Cell (Bio-Rad Laboratories, Inc., Hercules, CA, USA) at 20 volts for 20 min to transfer the proteins. Following the transfer, the membrane was blocked with BlockPRO™ Protein-Free Blocking Buffer (Visual Protein, Energenesis Biomedical Co., Ltd., Taipei, Taiwan) to prevent nonspecific binding. After overnight blocking, the membrane was washed with PBST (Visual Protein, Energenesis Biomedical Co., Ltd., Taipei, Taiwan) and incubated with a 1:10,000 dilution of 6× His Tag primary antibody (GTX115045, GeneTex, Inc., Hsinchu, Taiwan) for one hour on a shaker at 60 rpm. After washing with PBST, the membrane was incubated with a 1:10,000 dilution of Goat Anti-Rabbit IgG HRP-conjugated secondary antibody (GTX213110-01, GeneTex, Inc., Hsinchu, Taiwan) for one hour. Following a final wash with PBST, protein detection was performed using LumiFlash™ Ultima Chemiluminescent Substrate (Visual Protein, Energenesis Biomedical Co., Ltd., Taipei, Taiwan). The substrate was applied to the membrane, which was then immediately imaged using the iBright™ FL1500 Imaging System (Invitrogen™, Thermo Fisher Scientific Inc., Waltham, MA, USA). The recombinant protein, noted for its high productivity and purity, was selected as the antigen for the BEF subunit vaccine in subsequent experiments.

### 2.4. Vaccine Scheme

The recombinant protein with the highest productivity and purity was selected as the antigen for producing the BEF subunit vaccine with the adjuvant MONTANIDE™ ISA 206 VG (Seppic, La Garenne-Colombes, France) at a 1:1 ratio. The subunit vaccine, containing 150 μg of recombinant protein per dose, was injected subcutaneously into the necks of three cattle, three times, on Days 0, 14, and 28 to check its efficacy and safety. A sham vaccine was prepared using PBS and the same adjuvant in a 1:1 ratio and was injected subcutaneously into the necks of three cattle, following the same vaccination protocol as the sham group in this experiment. To ensure that the subunit vaccine was without any safety issue, local (redness, swelling, heat, or pain at the injection site) and systemic hypersensitive reactions (fever) were monitored by the veterinarian every day.

### 2.5. Animal Use and Ethics

To verify the efficacy of the recombinant protein, a subunit vaccine was produced and administered to three male Holstein cattle. The body weights of the experimental cattle ranged from 300 to 450 kg. The experimental cattle were checked for neutralizing antibody levels below 1:4 and appeared healthy with good body condition before vaccination. To refine the experiment and minimize the number of animals used in accordance with the 3R principles, the study included three cattle in the vaccination group and three cattle in the sham group. Blood samples for measuring neutralizing antibodies, cytokine expression, and BEFV detection were collected weekly by a licensed veterinarian. The experimental procedures involving animal use and blood sampling were approved by the Institutional Animal Care and Use Committee at National Pingtung University of Science and Technology, under the approval number 112-144.

### 2.6. Neutralization Test

Neutralizing antibodies and cytokine upregulation were two primary indicators used to assess vaccine efficacy in this experiment. Neutralizing antibody titers were measured weekly using a neutralization test. For this test, the BHK-21 cell line and quantified BEFV were employed. BEFV was quantified using the 50% Tissue Culture Infectious Dose (TCID_50_) assay, in which the virus was serially diluted tenfold in Dulbecco’s Modified Eagle Medium (Gibco™, Thermo Fisher Scientific Inc., Waltham, MA, USA) in duplicate and added to the BHK-21 cell line at 70–80% confluence. The cytopathic effect (CPE) of cells was stained with crystal violet, and the virus titer was determined after three days of incubation. For the neutralization test, BHK-21 cells were seeded in a 96-well plate the day before (Day 1) at a density of 1 × 10^4^ cells per well. Cattle serum samples were twofold serially diluted in duplicate and mixed with 100 TCID_50_ of BEFV. After incubating for one hour at 37 °C in a 5% CO_2_ incubator, the virus–serum mixtures were transferred to the BHK-21 cell plates and incubated at 37 °C in a 5% CO_2_ incubator until Day 5. The cells were stained with crystal violet, and neutralizing antibody titers were determined based on cell viability.

### 2.7. Cytokine Examination

In this study, IL-4, IL-10, IL-12B, IFN-γ, TNF-α, and β-actin were included for cytokine detection [21] (Table 1). The mRNA of various cytokine genes was extracted from buffy coat samples using the PureLink Viral RNA/DNA Mini Kit (Invitrogen™, Thermo Fisher Scientific Inc., Waltham, MA, USA) and reverse-transcribed into complementary DNA using the PrimeScript™ RT Reagent Kit (Takara Bio Inc., Shiga, Japan), following the manufacturer’s instructions. To measure cytokine expression at various time points in both vaccinated and sham groups, the mRNA from blood samples was analyzed using the SYBR Green™ system in real-time polymerase chain reaction (real-time PCR). All samples were tested in duplicate for each cytokine measurement.

Real-time PCR was performed using the QuantStudio™ 5 Real-Time PCR System (Applied Biosystems™, Thermo Fisher Scientific Inc., Waltham, MA, USA). The assays were conducted with PowerTrack™ SYBR Green Master Mix (Applied Biosystems™, Thermo Fisher Scientific Inc., Waltham, MA, USA) according to the manufacturer’s instructions. Reactions were prepared in a final volume of 20 μL, containing 1 μL of cDNA and 1 μL of each primer at the optimized concentration. Thermal cycling began at 50 °C for 2 min to degrade uracil-containing DNA, followed by 95 °C for 10 min to inactivate uracil-DNA glycosylase. Amplification proceeded with 40 cycles of 95 °C for 15 s, and 60 °C for 1 min for annealing and extension in a single step. Finally, the temperature was increased from 60 °C to 95 °C at a rate of 0.1 °C per second to generate the melting curve for each primer set.

All samples were analyzed in duplicate for each cytokine measurement. β-Actin served as the internal control for cytokine detection. The expression levels of each cytokine were quantified using the ΔCt and ΔΔCt methods. ΔCt was calculated by subtracting the cycle threshold (Ct) value of β-actin from that of the cytokine, while ΔΔCt was determined by comparing data collected on different days to the baseline measurement taken on the first day of vaccination (Day 0) for the same animal. The 2^−(ΔΔCt)^ value presents the fold change in cytokine gene expression in vaccinated or sham groups relative to Day 0 for each animal.

### 2.8. BEFV Detection

To ensure that no unexpected natural infections influenced the results, a real-time PCR assay using the TaqMan™ detection system was employed in this experiment. Two primer sets with probes for detecting BEFV and a bovine internal control (bIC) were designed (GeneReach Biotechnology Co., Taichung, Taiwan) and labeled with different fluorophores by the biotechnology company (Genomics Bioscience & Technology Co. Ltd., New Taipei, Taiwan). A relatively conserved region of the BEFV glycoprotein gene was selected as the target for BEFV detection, and the probe was labeled with a FAM reporter and an MGB quencher. A conserved mitochondrial region was chosen as the internal control of bovine origin (bIC), and the probe was labeled with a VIC reporter and an MGB quencher (Table 2). The TaqMan™ Fast Advanced Master Mix (Applied Biosystems™, Thermo Fisher Scientific Inc., Waltham, MA, USA) and QuantStudio™ 5 Real-Time PCR System (Applied Biosystems™, Thermo Fisher Scientific Inc., Waltham, MA, USA) were used in this experiment.

Primers and probes were diluted to 2 μM using ultrapure water and stored at −20 °C for subsequent analysis. To prepare the real-time PCR mixture, 10 μL of Master Mix, 0.5 μL of primers and probe, 2 μL of DNA, and ultrapure water were combined to a final volume of 20 μL. The amplification reaction was performed as follows: 50 °C for 2 min to degrade uracil-containing DNA, followed by 95 °C for 10 min to inactivate uracil-DNA glycosylase during the hold stage, then 40 cycles of 95 °C for 15 s, and 60 °C for 1 min to amplify the target genes. All samples were tested in duplicate, with both positive and negative controls included.

### 2.9. Statistical Analysis

Data describing differences between the two groups were analyzed with the Wilcoxon Two-Sample Test and differences were considered to be significant at a *p*-value < 0.05. All data were analyzed by JMP^®^ software (Student Edition 18, SAS Institute Inc., Cary, NC, USA).

## 3. Results

### 3.1. Recombinant Protein Produced by CHO Cell Expression System

**Figure 2:**
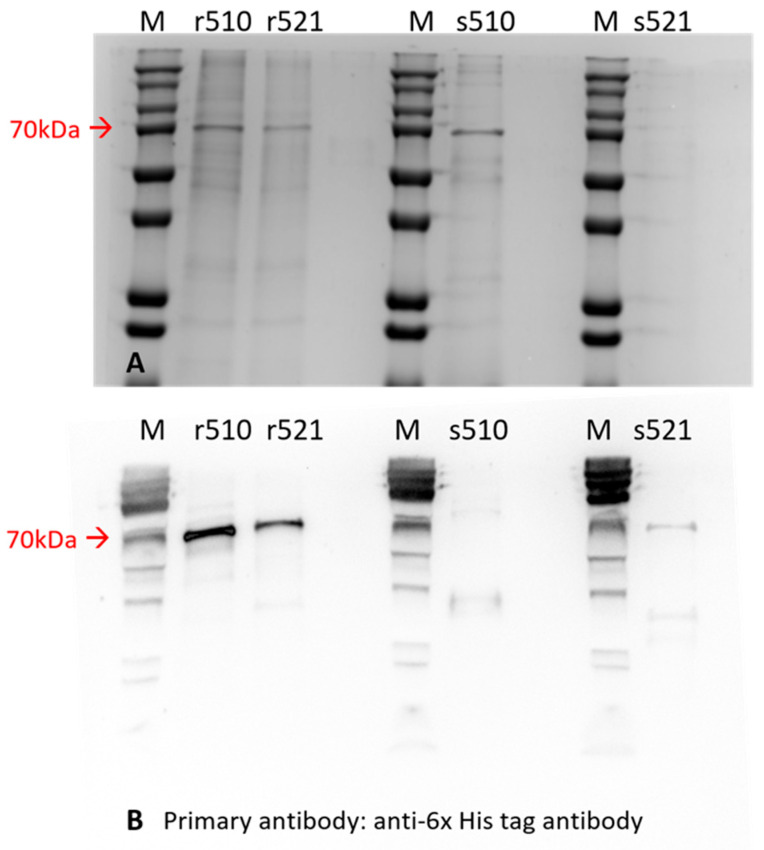
Recombinant proteins are shown in an SDS-PAGE gel (**A**) and on a PVDF membrane (**B**). All recombinant proteins in this study were present in the 10% SDS-PAGE gel after electrophoresis and on the 0.22 μm PVDF membrane after the Western blotting assay. Three out of four recombinant proteins (r510, r521, and s510) were successfully produced at the correct size of 70 kilodaltons, as shown in (**A**). Protein verification was performed using an anti-6× His Tag primary antibody for all expressed proteins in the Western blotting assay, as shown in (**B**). All four proteins were recognized by the anti-6X His Tag antibody in (**B**). M—Protein marker, kDa—kilo Dalton.

**Figure 3:**
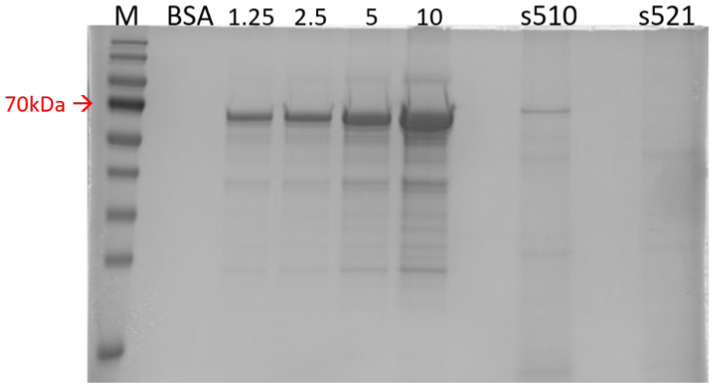
A protein quantification assay was conducted in the extrapolation test by comparing the concentration of recombinant proteins to standard BSA in a 10% SDS-PAGE gel. Protein s510 exhibited the highest yield and purity among the four recombinant proteins. M—Protein marker, BSA—Bovine serum albumin, kDa—kilo Dalton.

In this study, three of the four recombinant proteins were successfully generated at adequate levels using the CHO cell expression system (Figure 2). Specifically, proteins r510, r521, and s510 were produced at the expected size of 70 kilodaltons (kDa), as shown in the 10% SDS-PAGE gel in Figure 2A. Protein verification was performed using an anti-6× His Tag primary antibody for all expressed proteins in the Western blotting assay, as shown in Figure 2B. All four proteins were recognized by the anti-6× His Tag antibody in Figure 2B. Protein yield was estimated in the extrapolation test by comparing the concentration of recombinant proteins to standard BSA in the 10% SDS-PAGE gel (Figure 3). The yields were as follows: 20 mg/L for r510, 15 mg/L for r521, 30 mg/L for s510, and less than 10 mg/L for s521.

Regarding productivity and purity, protein s510 exhibited the highest yield and purity among the four recombinant proteins, reaching 30 mg/L in the culture supernatant of CHO cells, with a purity of approximately 80%. In contrast, protein s521 demonstrated poor productivity, yielding less than 10 mg/L, making it unsuitable as a candidate for subunit vaccine development in further experiments.

### 3.2. Subunit Vaccine Verification

#### 3.2.1. Neutralizing Antibody Titers Were Significantly Increased After Vaccination

**Figure 4:**
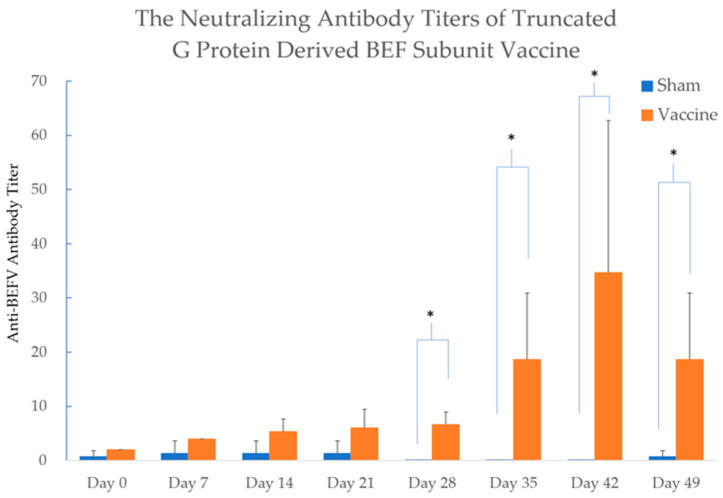
The titer of neutralizing antibody in the vaccinated and sham groups. In the vaccinated group, the average neutralizing antibody titer peaked at 35-fold the anti-BEFV antibody level by Day 42 of the experimental period. In contrast, few low antibody titers were detected in the sham group throughout the experimental period. The neutralizing antibody titers of the vaccinated groups showed significant differences on Days 28, 35, 42, and 49 compared to the sham groups. The differences between two groups were analyzed with the Wilcoxon Two-Sample Test. Significant differences (*p*-value < 0.05) were labeled with star marks. Glycoprotein is abbreviated as G Protein, and Bovine Ephemeral Fever is abbreviated as BEF in this figure.

The recombinant protein s510, which demonstrated the highest yield and purity, was produced as a subunit vaccine and administered to cattle three times at 14-day intervals. In the vaccinated group, the average neutralizing antibody titer peaked at 35-fold the anti-BEFV antibody level by Day 42 of the experimental period. In contrast, few low antibody titers were detected in the sham group throughout the experimental period. The neutralizing antibody titers of the vaccinated groups showed significant differences on Days 28, 35, 42, and 49 compared to the sham groups, with a *p*-value < 0.05 (Figure 4). Moreover, a slight swelling (2 × 3 cm) was observed at the injection site in cattle A, but not in cattle B and cattle C in the vaccination group. No other local or systemic adverse reactions were observed throughout the experimental period.

#### 3.2.2. A Significant Upregulation of IFN-γ Was Observed in the Vaccinated Group on Day 7 and Day 21

**Figure 5:**
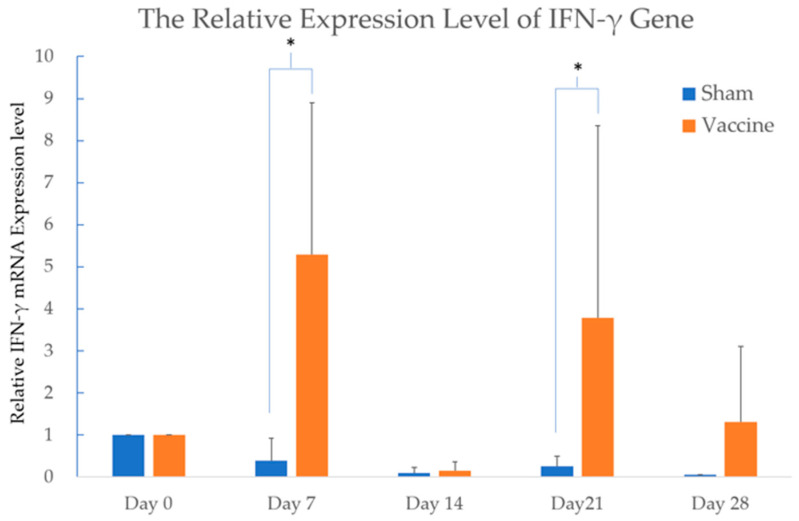
The expression of IFN-γ in both the vaccinated and sham groups. The value of IFN-γ gene expression is presented as 2^−(ΔΔCt)^ relative to Day 0. The subunit or sham vaccine was administered to each cattle on Days 0, 14, and 28. The expression of the IFN-γ gene in the vaccinated group showed a significant difference on Day 7 and Day 21 compared to the sham group. The differences between two groups were analyzed with the Wilcoxon Two-Sample Test. Significant difference (*p*-value < 0.05) is labeled with a star.

To measure cytokine expression at various time points in both the vaccinated and sham groups, the mRNA from blood samples was analyzed using the SYBR Green™ system in real-time PCR. The subunit vaccine was administered to each cattle on Days 0, 14, and 28, along with the peaks of IFN-γ gene upregulation that occurred 7 days after vaccination. The expression levels of the cytokines IL-4, IL-10, IL-12B, and TNF-α did not show any significant differences between the groups in this study. The expression of the IFN-γ gene in the vaccinated group showed a significant difference on Day 7 and Day 21 compared to the sham group, with a *p*-value < 0.05 (Figure 5).

#### 3.2.3. No BEFV Was Detected During the Experimental Period

**Figure 6:**
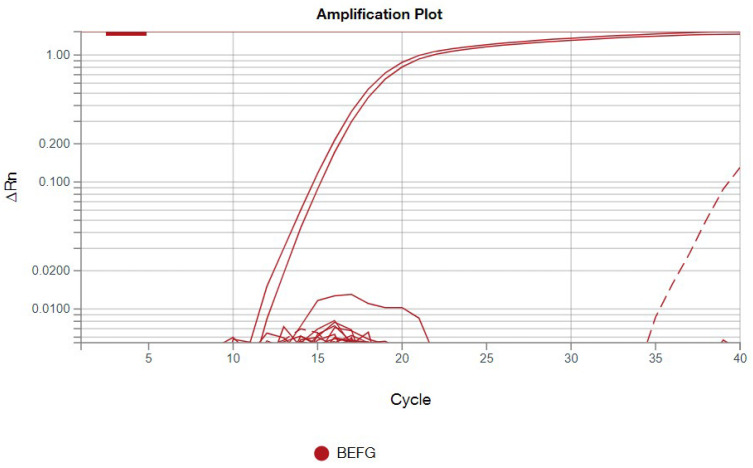
The results of the BEFV detection system are shown. The red solid line represents the positive control in duplicate. The red dotted line corresponds to cattle B in the sham group, with a Ct value of 39.8 in this experiment.

**Figure 7:**
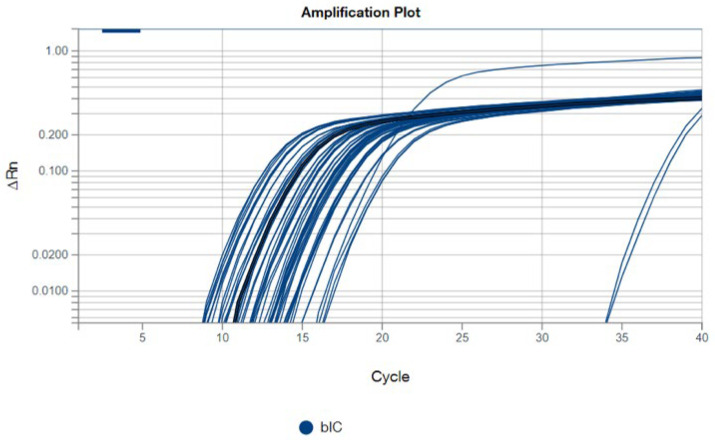
The results of the bovine-origin in BEFV detection system as the internal control (bIC). All samples except the negative control tested positive in this experiment.

For BEFV detection using the TaqMan™ system in real-time PCR, all samples were tested in duplicate, with both positive and negative controls included. The positive control exhibited a cycle threshold (Ct) value of 15.3 for BEFV detection. Aside from the positive control, only one sample from cattle B in the sham group showed a weak positive result, with a Ct value of 39.8, which was observed only once in the duplicate tests (Figure 6). For the internal control of bovine origin, all samples except the negative control tested positive (Figure 7), indicating the successful development of the BEFV and bovine-origin detection system in this study.

## 4. Discussion

In this study, a secretory truncated BEF G protein, s510, was successfully produced with a yield of 30 mg/L. It retained its antigenicity in cattle, as evidenced by increased neutralizing antibody titers when used as the antigen in an experimental subunit vaccine. The pcDNA3.4 vector is a constitutive mammalian expression system designed for high-level transgene expression in mammalian cells. It features a strong native CMV promoter and a Woodchuck Posttranscriptional Regulatory Element, both of which enhance gene expression efficiency. Due to limited space, only three cattle were included in each group for this experiment to verify the efficacy of the experimental subunit vaccine. The significant increase in neutralizing antibody levels and upregulation of the IFN-γ gene in these three cattle were sufficient to demonstrate the antigenicity of this novel truncated BEF G protein, which was the main objective of this study. However, the commercial use of this experimental subunit vaccine at the field level remains uncertain. Future studies on vaccine stability and the identification of an appropriate adjuvant are needed. After evaluating these parameters, a larger number of experimental cattle will be required for field testing or for viral challenge tests to assess the efficacy of the vaccine.

The amino acid sequence of protein s510 is located on the ectodomain of the G protein and includes all neutralizing antigenic sites from G1 to G4, although some parts of the ectodomain near the C-terminus were trimmed. The reason for truncating the protein is to increase protein productivity in cell culture. The rationale for truncating amino acids near the C-terminus is that antigenic sites G1 and G4 are linear neutralizing sites located at the C-terminal domain of the protein, whereas G2 and G3 are conformational neutralizing sites located at the N-terminal domain [18]. Linear neutralizing sites are considered more stable than conformational sites because they interact less with surrounding amino acids, allowing them to better maintain their structure and antigenicity. The results of the neutralization test in this study also support truncation of the C-terminus of the BEF G protein as an effective strategy, with neutralizing antibody titers reaching an average 35-fold increase in the vaccinated cattle. This level is considered sufficient to provide adequate protection against BEF infection in cattle [22].

There are varying and sometimes conflicting results regarding the protective neutralizing antibody titers required to prevent BEF infection. In the study by Vanselow et al., neutralizing antibody titers exceeding 1:45 after vaccination were associated with protection against developing clinical disease. However, in a subsequent study, one cattle with a neutralizing antibody titer of 1:64 still developed the clinical syndrome of BEF [23,24]. Based on a large-scale retrospective study of BEF occurrence and neutralizing antibody titers in cattle populations from 2001 to 2014 in Taiwan, neutralizing antibody titers below 1:32 were associated with clinical disease during BEF outbreaks, which is consistent with the findings of Wang et al. [10,22]. Therefore, according to these studies, a neutralizing antibody titer greater than 1:32 is considered protective against BEF infection in cattle.

Although no current research has administered a BEFV subunit vaccine in cattle, a dose of 50 μg of recombinant protein per animal was used in a Rift Valley Fever Virus glycoprotein subunit vaccine and successfully protected cattle against viremia [25]. According to their research, the dose of recombinant protein may be reduced in future vaccine dose-dependent efficacy tests. The peak of neutralizing antibody titer was observed in experimental cattle by Day 42, two weeks after the third vaccination. This effect may be attributed to subcutaneous administration route of vaccine administration used in this study. Subcutaneous administration is associated with a longer retention time at the injection site compared to intramuscular injection, which may prolong the plateau phase of neutralizing antibody titers. Additionally, subcutaneous injection is generally associated with less tissue irritation and may reduce local adverse reactions at the injection site, making it a safer option than intramuscular injection.

Among all the cytokines measured in this study, the significant upregulation of IFN-γ on Day 7 and Day 21 indicates effective antigen stimulation of the host immune system by this experimental subunit vaccine, supporting the efficacy of the s510-based subunit vaccine investigated here. The expression of the IFN-γ gene strongly correlates with IFN-γ protein expression in human peripheral blood mononuclear cells, as reported in 2022 [26]. Additionally, the SYBR Green real-time PCR assay has been widely used to examine IFN-γ gene expression and investigate immune responses in cattle [21]. Therefore, the upregulation of the IFN-γ gene after vaccination indicates a reliable immune response elicited by this vaccine. This increased expression of IFN-γ reflects the activation of T cells and natural killer cells, which are crucial for the immune response against intracellular pathogens such as viruses [27]. However, the upregulation of IFN-γ does not provide clear evidence of the specific types of immune cells in the host immune system. Using a flow cytometry assay is an effective way to determine which types of cells are involved in this immune reaction after vaccination. Moreover, a slight swelling (2 × 3 cm) was noted at the injection site in cattle A following vaccination, and a greater upregulation of IFN-γ was observed in the same animal. This suggests individual variability in immune response to this vaccine, which remains within the normal range of expected reactions. Combining the results of neutralizing antibody titers and the upregulation of IFN-γ levels indicates that the recombinant protein s510 holds promise as a novel antigen for the development of future BEF subunit vaccines.

Additionally, in this study, a BEFV detection system using the TaqMan™ system in a real-time PCR assay was successfully developed. This system can simultaneously detect BEFV and a bovine-origin gene in a single tube. It includes an internal control to detect bovine-derived elements, ensuring the accuracy of the sampling process and DNA extraction steps throughout the experiment. The high sensitivity is a key feature of this detection system. After quantitative validation, this system may be applied as a fast screening tool for detecting BEFV infection in the field.

## 5. Conclusions

This study represents significant progress in addressing the gap in evaluating the efficacy of recombinant BEF G protein-based subunit vaccines in cattle, which has been hindered by the low productivity of recombinant protein and the challenges of obtaining cattle for experimental conditions in previous studies. The main limitation of this study is the small number of animals, which reduces the statistical power and generalizability of the findings. In conclusion, the results indicate that the recombinant protein s510 is a promising antigen for future BEF subunit vaccine development in cattle, based on the combined neutralizing antibody titers and cytokine expression levels observed in this study.

## Figures and Tables

**Table 1:** The primer sets for cytokine detection were used with SYBR Green Master Mix in real-time PCR.

Cytokines	Primer Sequence	Optimal Final Concentration	TM (°C)
IL-4 F	CAGCATGGAGCTGCCT	300 nM	55.4
IL-4 R	ACAGAACAGGTCTTGCTTGC	300 nM	55.7
IL-10 F	CTTTAAGGGTTACCTGGGTTGC	300 nM	55.9
IL-10 R	CTCACTCATGGCTTTGTAGACAC	300 nM	55.5
IL-12B F	CAGCAGAGGCTCCTCTGAC	300 nM	57.5
IL-12B R	GTCTGGTTTGATGATGTCCCTG	300 nM	55.6
IFN-γ F	CAGAGCCAAATTGTCTCCTTC	600 nM	53.6
IFN-γ R	ATCCACCGGAATTTGAATCAG	600 nM	53
TNF-α F	CCAGAGGGAAGAGCAGTCC	300 nM	57.3
TNF-α R	GGCTACAACGTGGGCTACC	300 nM	58.3
B actin F	TGGGCATGGAATCCTG	600 nM	51.7
B actin R	GGCGCGATGATCTTGAT	600 nM	52.6

**Table 2:** The detail of primer sets for BEFV detection were used with TaqMan Master Mix in real-time PCR.

Primer Name	Sequence	Remark
BEFG F	AGAGCTTGGTGTGAATACAGA	
BEFG R	TTCCTCCTGCTGGTGCTGT	
BEFG P	TCAATATAGCCATCTTCATTCTT	3->MGB 5->FAM
bIC F	CACCAAGAGAATCAAGCACGAA	
bIC R	TGCGTGCTTCATGGCCTAA	
bIC P	CTTAGTTTACTGCTAAATCCT	3->MGB 5->VIC

## Data Availability

No new data were available for this publication.
